# Genetic dissection of anterior segment dysgenesis caused by a *Col4a1* mutation in mouse

**DOI:** 10.1242/dmm.027888

**Published:** 2017-04-01

**Authors:** Mao Mao, Márton Kiss, Yvonne Ou, Douglas B. Gould

**Affiliations:** 1 Department of Ophthalmology, Institute for Human Genetics, UCSF School of Medicine, San Francisco, CA 94143, USA; 2 Department of Genetics, University of Szeged, Középfasor 52, Szeged H-6726, Hungary; 3 Department of Anatomy, Institute for Human Genetics, UCSF School of Medicine, San Francisco, CA 94143, USA

**Keywords:** Anterior segment dysgenesis, Basement membrane, COL4A1, Mouse

## Abstract

Ocular anterior segment dysgenesis (ASD) describes a spectrum of clinically and genetically heterogeneous congenital disorders affecting anterior structures that often lead to impaired vision. More importantly, 50-75% of patients with ASD develop early onset and aggressive glaucoma. Although several genes have been implicated in the etiology of ASD, the underlying mechanisms remain elusive. Type IV collagen alpha 1 (COL4A1) is an extracellular matrix protein and a critical component of nearly all basement membranes. *COL4A1* mutations cause multi-system disorders in patients, including ASD (congenital cataracts, Axenfeld-Rieger's anomaly, Peter's anomaly and microphthalmia) and congenital or juvenile glaucoma. Here, we use a conditional *Col4a1* mutation in mice to determine the location and timing of pathogenic events underlying COL4A1-related ocular dysgenesis. Our results suggest that selective expression of the *Col4a1* mutation in neural crest cells and their derivatives is not sufficient to cause ocular dysgenesis and that selective expression of the *Col4a1* mutation in vascular endothelial cells can lead to mild ASD and optic nerve hypoplasia but only on a sensitized background. In contrast, lens-specific expression of the conditional *Col4a1* mutant allele led to cataracts, mild ASD and optic nerve hypoplasia, and age-related intraocular pressure dysregulation and optic nerve damage. Finally, ubiquitous expression of the conditional *Col4a1* mutation at distinct developmental stages suggests that pathogenesis takes place before E12.5. Our results show that the lens and possibly vasculature play important roles in *Col4a1*-related ASD and that the pathogenic events occur at mid-embryogenesis in mice, during early stages of ocular development.

## Abstract (precis)

**Summary:** Key pathogenic events in anterior segment dysgenesis, a congenital ocular disease with complex etiology, are recapitulated in a mouse model of *Col4a1*-related ASD.

## INTRODUCTION

Anterior segment dysgenesis (ASD) describes a spectrum of ocular developmental disorders affecting structures located in the front of the eye, including the cornea, iris, lens, ciliary body and ocular drainage structures (trabecular meshwork and Schlemm's canal) (Gould and John, 2002). Clinical manifestations of ASD are variable and include cataracts, corneal opacities, misshapen pupils, iris hypoplasia and iridocorneal adhesions (synechia), and can also present as syndromes associated with extraocular defects (Ito and Walter, 2014). ASD can acutely impair vision by obstructing the light path through the cornea and lens en route to the photoreceptors in the outer retina or lead to progressive vision loss when dysmorphic drainage structures lead to insufficient outflow of aqueous humor, elevated intraocular pressure (IOP) and eventual death of retinal ganglion cells. Notably, approximately 50-75% of patients with ASD develop high IOP and irreversible vision loss due to glaucoma (Alward, 2000; Waring et al., 1975).

Ocular anterior segment development involves a series of highly orchestrated and complex inductive interactions between tissues derived from four embryonic lineages: the surface ectoderm, neural ectoderm and the periocular mesenchyme derived from neural crest and cranial paraxial mesoderm (Trainor and Tam, 1995; Gage et al., 2005). In mice, the optic vesicle (neural ectoderm) induces lens placode (surface ectoderm) formation around embryonic day (E)9.5 after which the prospective lens invaginates and buds off from the ectodermal tissue fated to become the corneal epithelium. By E10.5, the periocular mesenchyme, which originates from the neural crest and paraxial mesoderm, migrates between the lens vesicle and surface ectoderm and differentiates to generate corneal endothelium and stroma, iris stroma, ciliary body muscles, trabecular meshwork and Schlemm's canal (Gage and Zacharias, 2009; Gould et al., 2004). Thus, the tissues commonly affected in ASD mainly arise from the periocular mesenchyme, which is predominantly derived from the neural crest.

ASD is a multigenic disorder, and allelic differences and genetic background have been shown to contribute to clinical heterogeneity (Gould and John, 2002). It is well established that mutations in *CYP1B1*, *FOXC1*, *FOXC2*, *FOXE3*, *LMX1B*, *MAF*, *PAX6*, *PITX2* and *PITX3* (Reis and Semina, 2011; Ito and Walter, 2014) cause ASD. Many of these genes encode transcription factors expressed in the lens or periocular mesenchyme (Sowden, 2007). Of these, mutations in *FOXC1* and *PITX2* are estimated to be the underlying genetic cause in 40% of patients with ASD (D'Haene et al., 2011). Although the importance of *FOXC1* and *PITX2* has long been recognized, their precise roles in ocular development and the mechanism(s) by which mutations in these genes cause ASD remain to be fully elucidated (Acharya et al., 2011; Tamimi et al., 2004; Sommer et al., 2006; Paylakhi et al., 2013; Chen et al., 2016; Kumar and Duester, 2010; Hjalt et al., 2001). Recently, mutations in genes encoding proteins associated with the extracellular matrix, including *LAMB2* (Zenker et al., 2004), *B3GALTL* (Lesnik Oberstein et al., 2006), *PXDN* (Khan et al., 2011) and *COL4A1* (Sibon et al., 2007; Rødahl et al., 2013; Shah et al., 2012; Coupry et al., 2010) have also been identified in patients with ASD and developmental glaucoma. However, even less is known about the pathogenic mechanisms by which mutations in these genes cause ASD.

Independent mutagenesis screens in mice showed that mutations in the genes encoding collagen, type IV, alpha 1 (COL4A1) and alpha 2 (COL4A2) cause ASD (Gould et al., 2005; Van Agtmael et al., 2005; Favor et al., 2007). We previously identified a semi-dominant splice site mutation resulting in deletion of exon 41 (*Col4a1^Δex41^*) that leads to embryonic lethality in homozygous mutant mice and reduced viability in heterozygous mutant mice (Gould et al., 2005). Animals that survive develop highly penetrant ASD and optic nerve hypoplasia (ONH) as part of a multi-system disorder that also includes cerebrovascular, muscular and renal defects (Gould et al., 2007; Labelle-Dumais et al., 2011; Mao et al., 2015a). Analyses of developing eyes from *Col4a1^+/Δex41^* mice revealed embryonic anterior chamber hyphema, iridocorneal adhesions and dysgenesis of the ocular drainage structures and ciliary body, as well as open pupil, cataract, iris vasculature tortuosity and anterior chamber enlargement (Gould et al., 2007). Subsequently, *COL4A1* mutations were identified in patients with a spectrum of ocular defects including ONH (Labelle-Dumais et al., 2011; Yoneda et al., 2013), microphthalmia (Deml et al., 2014; Yoneda et al., 2013), cataract (Kuo et al., 2012; Xia et al., 2014), microcornea (Coupry et al., 2010), Axenfeld-Rieger's anomaly (Coupry et al., 2010; Sibon et al., 2007; Rødahl et al., 2013), Peter's anomaly (Deml et al., 2014), high IOP (Sibon et al., 2007) and glaucoma (Deml et al., 2014; Giorgio et al., 2015; Plancher et al., 2015; Meuwissen et al., 2015).

Heterotrimers composed of two COL4A1 peptides and one COL4A2 peptide assemble in the endoplasmic reticulum (ER) before being secreted into basement membranes where they polymerize to form an intricate network that interacts with other extracellular and cell surface proteins (Trüeb et al., 1982; Mayne et al., 1984). The majority of *COL4A1* and *COL4A2* mutations occur in the triple helical domain and appear to impair heterotrimer secretion (Jeanne and Gould, 2016). While there are important allelic differences, heterotrimers that incorporate mutant COL4A1 generally accumulate within the ER at the expense of secretion (Kuo et al., 2012). COL4A1/A2 heterotrimers are major components of nearly all basement membranes including those of the developing eye (Bai et al., 2009). As ASD is a developmental disorder, the first step in understanding the molecular mechanism(s) by which *COL4A1* and *COL4A2* mutations cause ocular disease is to define the spatial and temporal parameters of early pathogenic events. To this end, we employed a conditional inducible approach to express mutant *Col4a1* in cells of different embryonic lineages or at different stages of development. Our findings identify important contributions of the lens for both cell-autonomous and cell non-autonomous aspects of *Col4a1*-related ASD and suggest a potential role for vascular endothelial cells in the etiology of disease. Furthermore, we show that the key pathogenic events may take place during early stages of ocular development.

## RESULTS

### Ubiquitous expression of a conditional *Col4a1* mutant allele recapitulates the ASD phenotypic spectrum observed in *Col4a1^+/Δex41^* mice

**Fig. 1.**
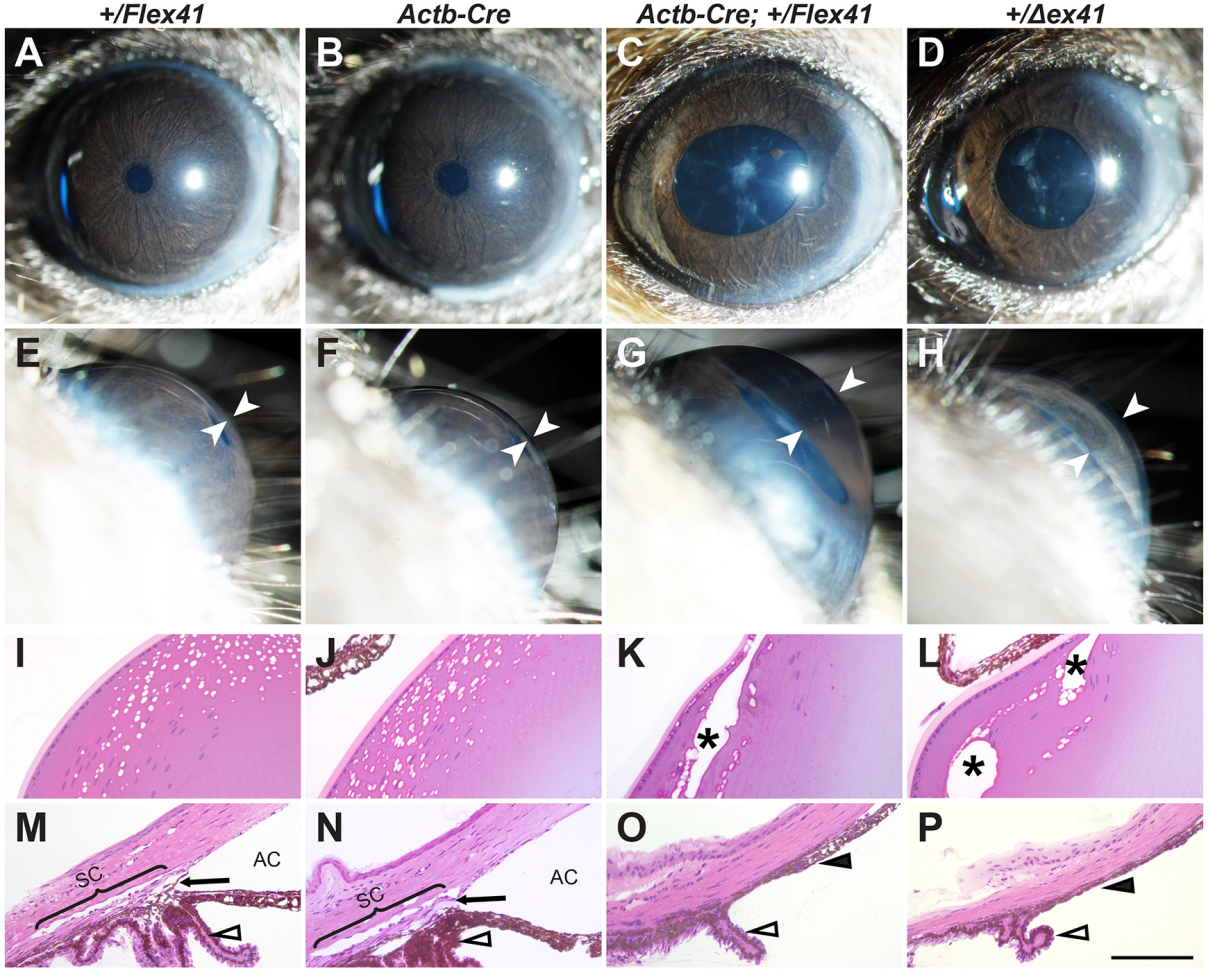
**Ubiquitous recombination of the *Col4a1^Flex41^* allele recapitulates the full phenotypic spectrum of *Col4a1^+/Δex41^* ASD.** (A-H) *Col4a1^Flex41^* mice were crossed to the ubiquitous *Actb^Cre^* strain and the progeny were analyzed by slit lamp biomicroscopy at 1.0-1.5 months of age (*n*=24, 38 and 26 eyes for *Col4a1^+/Flex41^*, *Actb^Cre^* and *Actb^Cre^;Col4a1^+/Flex41^* mice, respectively). To compare phenotypic severity, *Col4a1^+/Δex41^* mice were also included (*n*=32 eyes). While eyes from control (*Col4a1^+/Flex41^* and *Actb^Cre^*) animals were completely normal (A,B,E,F), *Actb^Cre^;Col4a1^+/Flex41^* mice (C,G) had abnormal iris vasculature, open pupil, pigment on the lens, cataracts and enlarged anterior chambers that were comparable to *Col4a1^+/Δex41^* mice (D,H). White arrowheads indicate anterior chamber. (I-L) Histological analysis of lens shows anterior subcapsular vacuoles (asterisks) in eyes from *Actb^Cre^;Col4a1^+/Flex41^* conditional (K) mutants that are similar to *Col4a1^+/Δex41^* mice (L) but not present in eyes from control mice (I,J) (*n*=5 eyes per genotype). (M-P) Histological analysis of iridocorneal angles in control mice (*Col4a1^+/Flex41^* and *Actb^Cre^,* M and N) at 1.0-1.5 months of age showing open anterior chambers (AC) and Schlemm's canals (SC; bracket) separated by trabecular meshwork (black arrows). Posterior to the iridocorneal angle, control animals show multifoliated ciliary bodies (open arrowhead). In contrast, both *Actb^Cre^;Col4a1^+/Flex41^* (O) and *Col4a1^+/Δex41^* (P) mutants have hypoplastic ciliary bodies and dysgenic iridocorneal angles without discernable Schlemm's canal or trabecular meshwork, accompanied by extensive iridocorneal adhesions (black arrowheads). Scale bar: 100 μm.

We have previously characterized ASD in mice with a *Col4a1* splice acceptor mutation that results in deletion of exon 41 (*Δex41*) (Gould et al., 2005, 2007; Mao et al., 2015b). We sought to generate an equivalent conditional *Col4a1* mutation with which to determine the location and timing of important pathogenic events underlying *Col4a1-*related ocular dysgenesis (Alavi et al., 2016; Jeanne et al., 2015). The original conditional allele that we tested had *loxP* sites flanking exon 41 and a neomycin selection cassette (*neo*) that was flanked by *Frt* sites (*Col4a1^+/Flex41-neo^*) (Fig. S1A). Surprisingly, even in the absence of *Cre* expression, ∼20% of eyes from *Col4a1^+/Flex41-neo^* mice had torturous, dilated iris vasculature and slightly enlarged anterior chamber – two common features of ASD seen in *Col4a1* mutant mice (Fig. S1B-E). In addition, cloudy corneas, cataracts and abnormally shaped pupils were occasionally observed by slit lamp examinations (Fig. S1F). Furthermore, intercrosses between *Col4a1^+/Flex41-neo^* mice failed to generate homozygous *Col4a1^Flex41-neo/Flex41-neo^* progeny, suggesting that pups with this genotype were not viable. When we attempted to remove the *neo* cassette by crossing *Col4a1^+/Flex41-neo^* with *Actb^Flp^* mice (Rodríguez et al., 2000) we were unable to produce double heterozygous progeny (*n*=0/127 pups). Therefore, we deleted the *neo* selection cassette from the embryonic stem cell clones to generate a new line with a ‘clean’ conditional allele (*Col4a1^Flex41^*). *Col4a1^+/Flex41^* mice have no discernible phenotype even when bred to homozygosity. We next crossed *Col4a1^+/Flex41^* mice to a ubiquitous Cre line (*Actb^Cre^*) to validate the conditional *Col4a1* mutant allele and determine if it recapitulates the ASD phenotypic spectrum that we have described previously in *Col4a1^+/Δex41^* mice (Gould et al., 2007). We first confirmed the presence of the recombinant form of *Col4a1* mRNA in eyes from *Actb^Cre^;Col4a1^+/Flex4^*^1^ mice by RT-PCR (Fig. S2A). Slit lamp examinations revealed no abnormalities in *Col4a1^+/Flex41^* or *Actb^Cre^* mice at 1.0-1.5 months of age (Fig. 1A-H). In contrast, ubiquitous conditional mutants (*Actb^Cre^;Col4a1^+/Flex4^*^1^) displayed the full ASD phenotypic spectrum, including dilated pupils, pigment dispersion, torturous and dilated iris vasculature, cataracts and enlarged anterior chambers. Moreover, the defects observed in *Actb^Cre^;Col4a1^+/Flex4^*^1^ mice were comparable to those described in *Col4a1^+/Δex41^* mice. To further characterize the ocular defects, we next performed histological analysis on eyes at 1.0-1.5 months (Fig. 1I-P). Eyes of both *Actb^Cre^;Col4a1^+/Flex4^*^1^ and *Col4a1^+/Δex41^* mice exhibited various ocular defects that were never observed in littermate controls (*Col4a1^+/Flex4^*^1^ and *Actb^Cre^*), including cataracts, iridocorneal adhesions, compressed or absent trabecular meshwork and Schlemm's canals, and dysgenic ciliary bodies (compare K,L with I,J, and O,P with M,N in Fig. 1). Together, these results confirmed that ubiquitous expression of the conditional *Col4a1^Flex4^*^1^ allele could functionally recreate the full ASD phenotypic spectrum observed in *Col4a1^+/Δex41^* mice.

### Selective expression of mutant COL4A1 in neural crest-derived periocular mesenchyme does not cause ASD

**Fig. 2.**
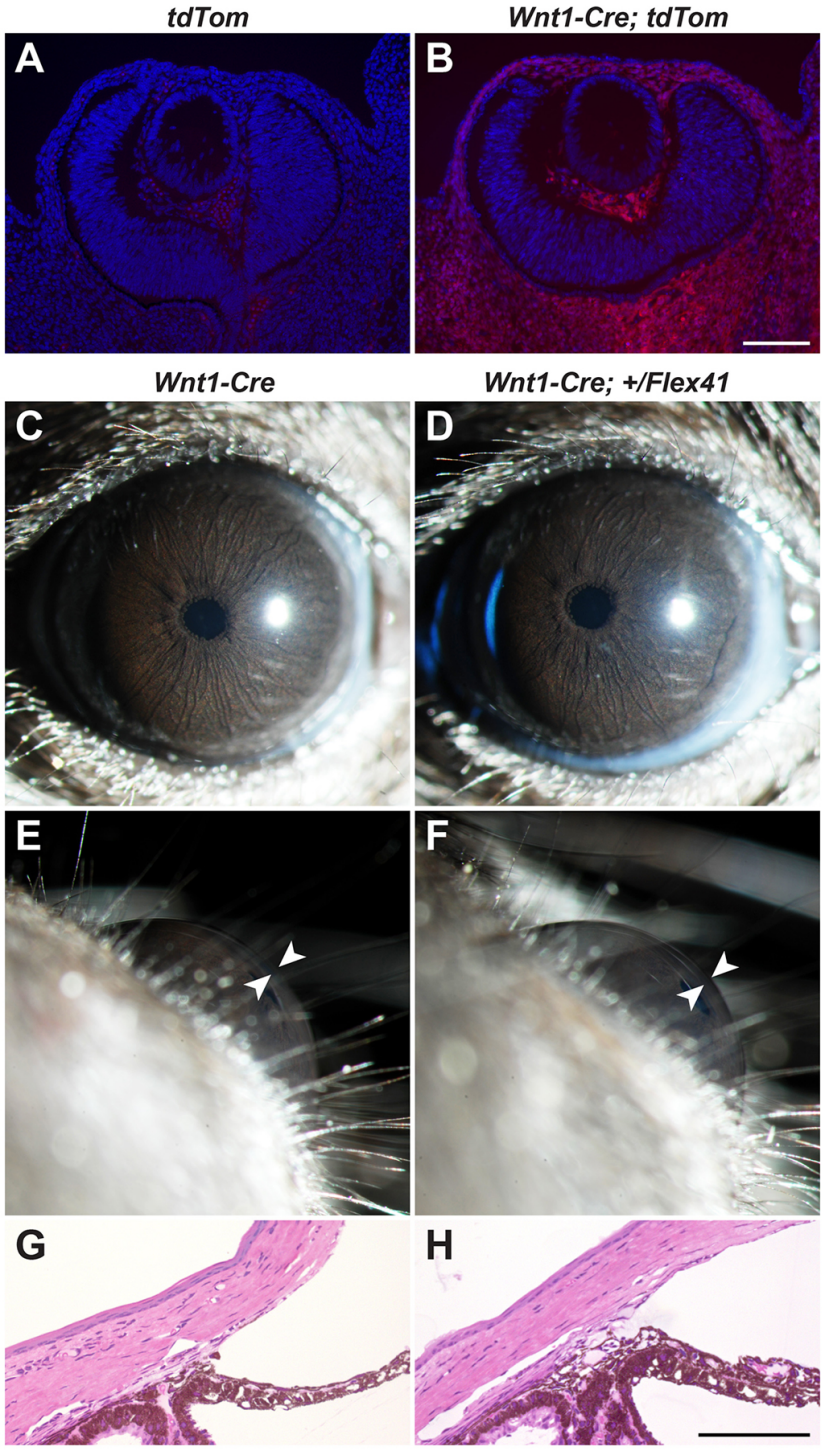
**Selective expression of mutant COL4A1 in neural crest-derived periocular mesenchyme does not cause ASD.** *Col4a1^+/Flex41^* mice were crossed to the *Wnt1^Cre^* strain to selectively express mutant COL4A1 protein in neural crest-derived periocular mesenchyme. (A,B) Validation of CRE expression in neural crest-derived periocular mesenchyme using a reporter line that expresses tdTomato after CRE-mediated recombination. Eyes were examined at E12.5. Scale bar: 100 μm. (C-F) Control (*Wnt1^Cre^*, *n*=22 eyes) and mutant (*Wnt1^Cre^;Col4a1^Flex41^*, *n*=24 eyes) eyes appeared to be normal by slit lamp biomicroscopic examination. Arrowheads indicate anterior chamber. (G,H) Histological analysis reveals no abnormalities of the iridocorneal angle or ciliary body (*n*=6 eyes for both genotypes). Scale bar: 100 μm.

Because anterior segment structures originate predominantly from neural crest-derived periocular mesenchyme (Gage et al., 2005), we tested if selective expression of the mutant *Col4a1* allele in neural crest cells and their derivatives would contribute to ASD. To this end, we crossed the conditional *Col4a1^Flex4^*^1^ allele to the *Wnt1^Cre^* line that expresses CRE recombinase in neural crest cells before they migrate from the neural tube. The *Wnt1^Cre^* line has been characterized previously (Danielian et al., 1998) and used in multiple studies (Martino et al., 2016; Iwao et al., 2009; Ittner et al., 2005), and we confirmed the presence of CRE activity in the periocular mesenchyme using a *Rosa26^tdTomato^* reporter line (Madisen et al., 2010) (Fig. 2A,B). Slit lamp biomicroscopy and histological analysis failed to reveal ocular abnormalities in *Wnt1^Cre^;Col4a1^+/Flex4^*^1^ mice (Fig. 2C-H), suggesting that dysgenesis of neural crest-derived anterior ocular structures in *Col4a1* mutant mice may be cell non-autonomous.

### Selective expression of mutant COL4A1 in vascular endothelium reveals a potential vascular contribution to ASD

**Fig. 3.**
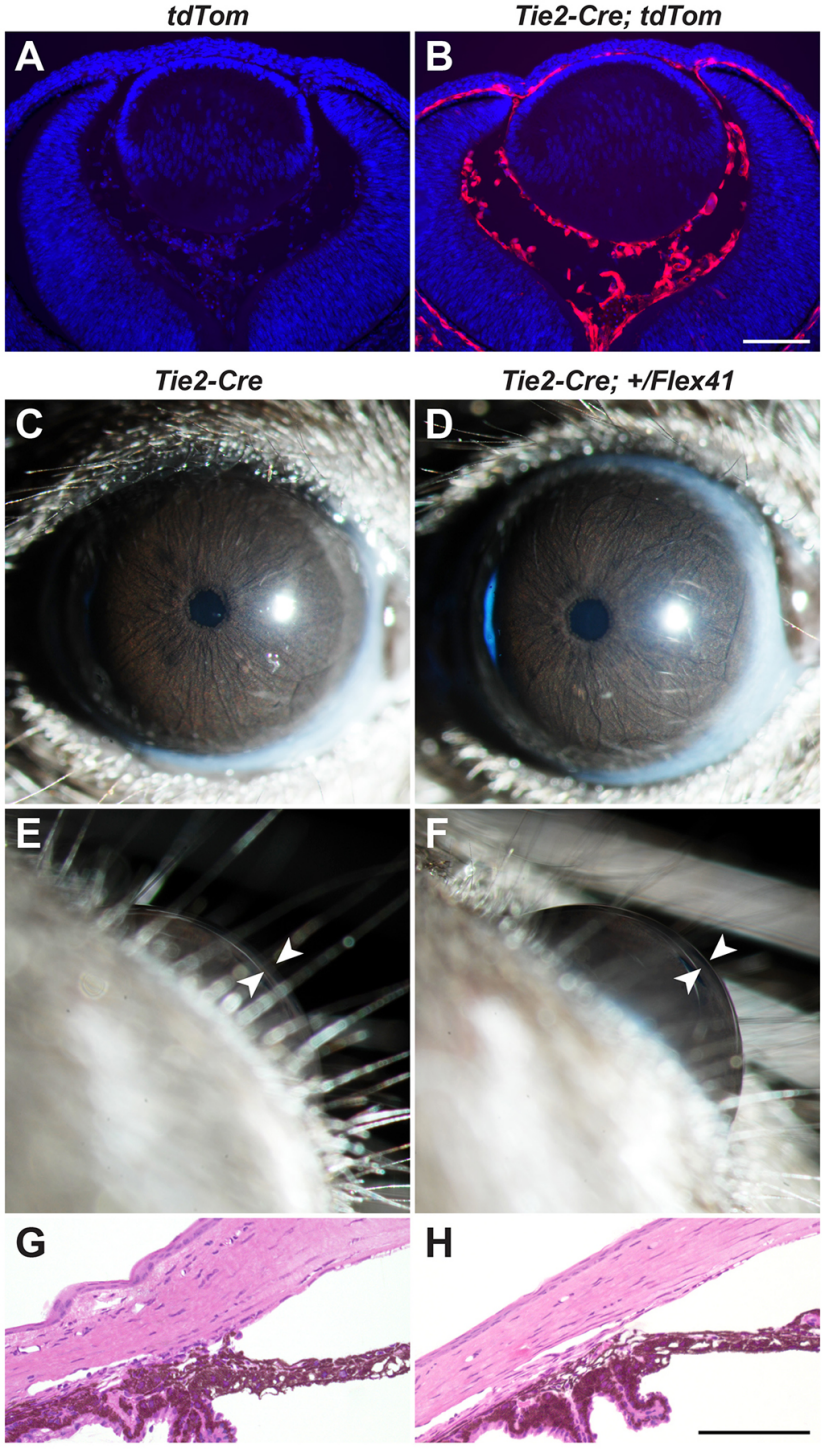
**Selective expression of mutant COL4A1 in vascular endothelial cells does not cause ASD.** (A,B) *Col4a1^+/Flex41^* mice were crossed to the *Tie2^Cre^* strain for selective expression of mutant COL4A1 protein in vascular endothelial cells (image of E12.5 eye). (C-F) Eyes from control (*Tie2^Cre^*, *n*=28 eyes) and mutant (*Tie2^Cre^;Col4a1^+/Flex41^*, *n*=22 eyes) mice appeared to be normal by slit lamp biomicroscopic examinations. Arrowheads indicate anterior chamber. (G,H) Histological analysis does not reveal any morphological abnormalities in the iridocorneal angle of eyes from control (*n*=4 eyes) or mutant (*n*=6 eyes) mice. Scale bar: 100 μm.

*Col4a1^+/Δex41^* mice have anterior chamber hyphema and abnormal iris vasculature, suggesting that there could be a vascular contribution to ASD (Gould et al., 2007). To test a role for the vasculature in ASD pathogenesis we used the *Tie2^Cre^* mouse line to selectively express mutant *Col4a1* in vascular endothelial cells (Fig. 3A,B). We did not detect ocular defects by slit lamp examination and histological analysis of *Tie2^Cre^;Col4a1^+/Flex41^* mice (Fig. 3C-H). This finding was in contrast to a previous observation of an ocular phenotype in similar mice with selective vascular endothelial cell expression of the conditional *Col4a1* mutant allele that retained the *neo* cassette (*Tie2^Cre^;Col4a1^+/Flex41-neo^*). Those mice had ASD and ONH that resembled pathology seen in *Col4a1^+/Δex41^* mice (Fig. S3). While cataracts and dilated pupils were seen less frequently in *Tie2^Cre^;Col4a1^+/Flex41-neo^* compared with *Col4a1^+/Δex41^* mice, slit lamp biomicroscopy revealed large and tortuous iris vessels, iridocorneal adhesions and slightly enlarged anterior chambers in the majority of *Tie2^Cre^;Col4a1^+/Flex41-neo^* eyes examined (Fig. S3A,B). In addition, histological analysis confirmed extensive iridocorneal adhesions and compressed or absent ocular drainage structures in all *Tie2^Cre^;Col4a1^+/Flex41-neo^* eyes examined (Fig. S3C,D) and significantly reduced optic nerve cross-sectional areas (Fig. S3E). Collectively, these findings suggest that the vasculature could contribute to COL4A1-mediated ASD in sensitized genetic contexts.

### Selective expression of mutant COL4A1 in the lens causes cell autonomous and cell non-autonomous phenotypes

**Fig. 4.**
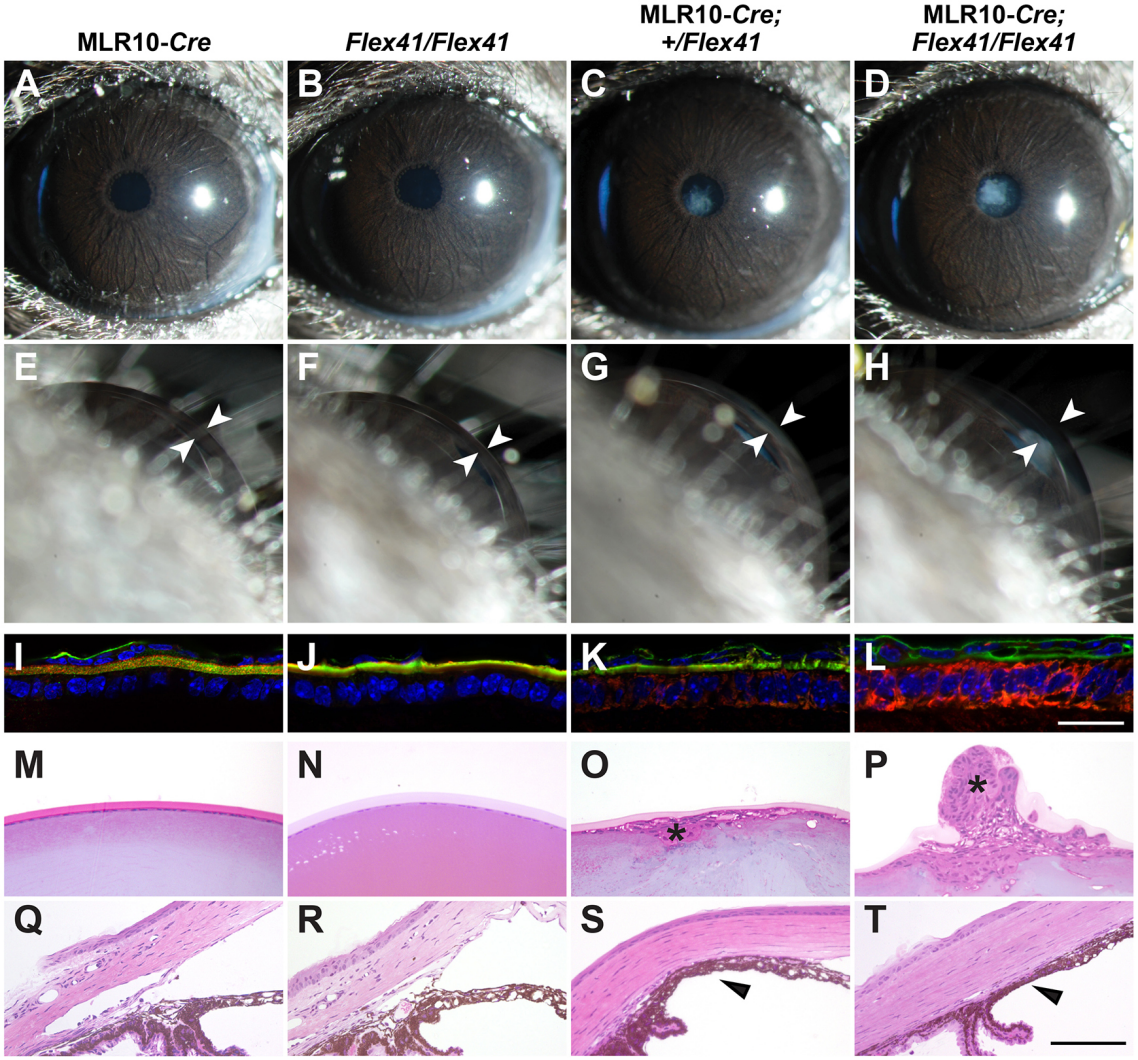
**Selective expression of mutant COL4A1 in lens leads to dose-dependent ASD.** *Col4a1^Flex41^* mice were crossed to the MLR10*^Cre^* strain for selective expression of mutant COL4A1 protein in the lens. (A-H) While control eyes (A,B,E,F) (*n*=14 and 22 eyes from MLR10*^Cre^* and *Col4a1^Flex41/Flex41^* mice, respectively) were normal upon slit lamp examination, cataracts are observed in lens-specific mutants (C,D,G,H) at 1.0-1.5 months of age (15/24 and 30/30 eyes from MLR10*^Cre^;Col4a1^+/Flex41^* and MLR10*^Cre^;Col4a1^Flex41/Flex41^*, respectively). In addition, a higher dosage of the *Col4a1* mutant allele (H) leads to a slight increase in anterior chamber depth (27/30 eyes in MLR10*^Cre^;Col4a1^Flex41/Flex41^*). White arrowheads indicate anterior chamber. (I-L) Mutant COL4A1 accumulates within cells at the expense of secretion into basement membranes. In control animals (MLR10*^Cre^* and *Col4a1^Flex41/Flex41^,* I and J), most COL4A1 immunolabeling (red) colocalizes with laminin (green) in the lens capsule. However, in the conditional (K,L) mutants (MLR10*^Cre^;Col4a1^+/Flex41^* and MLR10*^Cre^;Col4a1^Flex41/Flex41^*), increased COL4A1 immunolabeling is detected within lens epithelial cells (*n*=6 eyes per genotype). In addition, a dosage effect was observed with higher levels of intracellular COL4A1 accumulation detected in homozygous mutants (MLR10*^Cre^;Col4a1^Flex41/Flex41^*) compared with heterozygous mutants (MLR10*^Cre^;Col4a1^+/Flex41^*) (K compared with L). Scale bar: 20 μm. (M-P) Histological analysis reveals subcapsular abnormalities (asterisks) in lenses from mutant mice (*n*=5 for MLR10*^Cre^;Col4a1^+/Flex41^* and 11 for MLR10*^Cre^;Col4a1^Flex41/Flex41^*) but not control mice (*n*=10 for MLR10*^Cre^* and 9 for *Col4a1^Flex41/Flex41^*). (Q-T) The trabecular meshwork and Schlemm's canal appear normal in most sections from both heterozygous and homozygous mutants. However, focal abnormalities, including compressed drainage structures (3/5 and 5/11 eyes from heterozygous and homozygous mutants, respectively) and iridocorneal adhesions with obliteration of the angle (black arrowheads; 1/5 and 1/11 eyes from heterozygous and homozygous mutants, respectively) are seen. Scale bar: 100 μm.

The lens plays important roles in the inductive interactions required for ocular anterior segment development and primary lens defects can cause ASD (Beebe and Coats, 2000; Flügel-Koch et al., 2002). Moreover, the lens capsule, which surrounds the entire lens, is a prominent basement membrane that contains significant amounts of type IV collagen (Cummings and Hudson, 2014; Bai et al., 2009). To test whether primary lens defects contribute to *Col4a1*-related ASD we crossed *Col4a1^+/Flex4^*^1^ mice with MLR10*^Cre^* mice, which express CRE as early as E10.5 when the lens vesicle forms (Zhao et al., 2004). Slit lamp biomicroscopy of MLR10*^Cre^*;*Col4a1^+/Flex41^* mice revealed the presence of cataracts but no anterior chamber enlargement (Fig. 4A-C and E-G). To test whether there might be a ‘dose response’ to mutant COL4A1 we also generated and analyzed mice that were homozygous for the conditional allele (MLR10*^Cre^;Col4a1^Flex41/Flex41^*). When we compared intracellular retention of mutant COL4A1 in lens epithelial cells we saw a graded response with greater COL4A1 accumulation in MLR10*^Cre^;Col4a1^Flex41/Flex41^* compared with MLR10*^Cre^*;*Col4a1^+/Flex41^* mice (Fig. 4I-L). In contrast to heterozygotes, slit lamp examination of MLR10*^Cre^;Col4a1^Flex41/Flex41^* mice demonstrated mildly enlarged anterior chambers (Fig. 4H compared with G) in addition to cataracts (Fig. 4D) and histological analysis showed more severe disruptions of the lens including large plaques and focal anterior lens extrusions (Fig. 4M-P). Although morphology of the ocular drainage structures in the iridocorneal angle appeared to be relatively normal in most tissue sections, focal dysgenesis and iridocorneal adhesions were detected occasionally in MLR10*^Cre^*;*Col4a1^+/Flex41^* and MLR10*^Cre^;Col4a1^Flex41/Flex41^* eyes (Fig. 4S,T). Ocular defects were never observed in control *Col4a1^Flex41/Flex41^* and MLR10*^Cre^*;*Col4a1^+/+^* mice (Fig. 4M,N,Q and R). Taken together, these data suggest that the lens has both cell-autonomous and cell non-autonomous contributions to *Col4a1*-related ASD and that the effects are dose dependent.

**Fig. 5.**
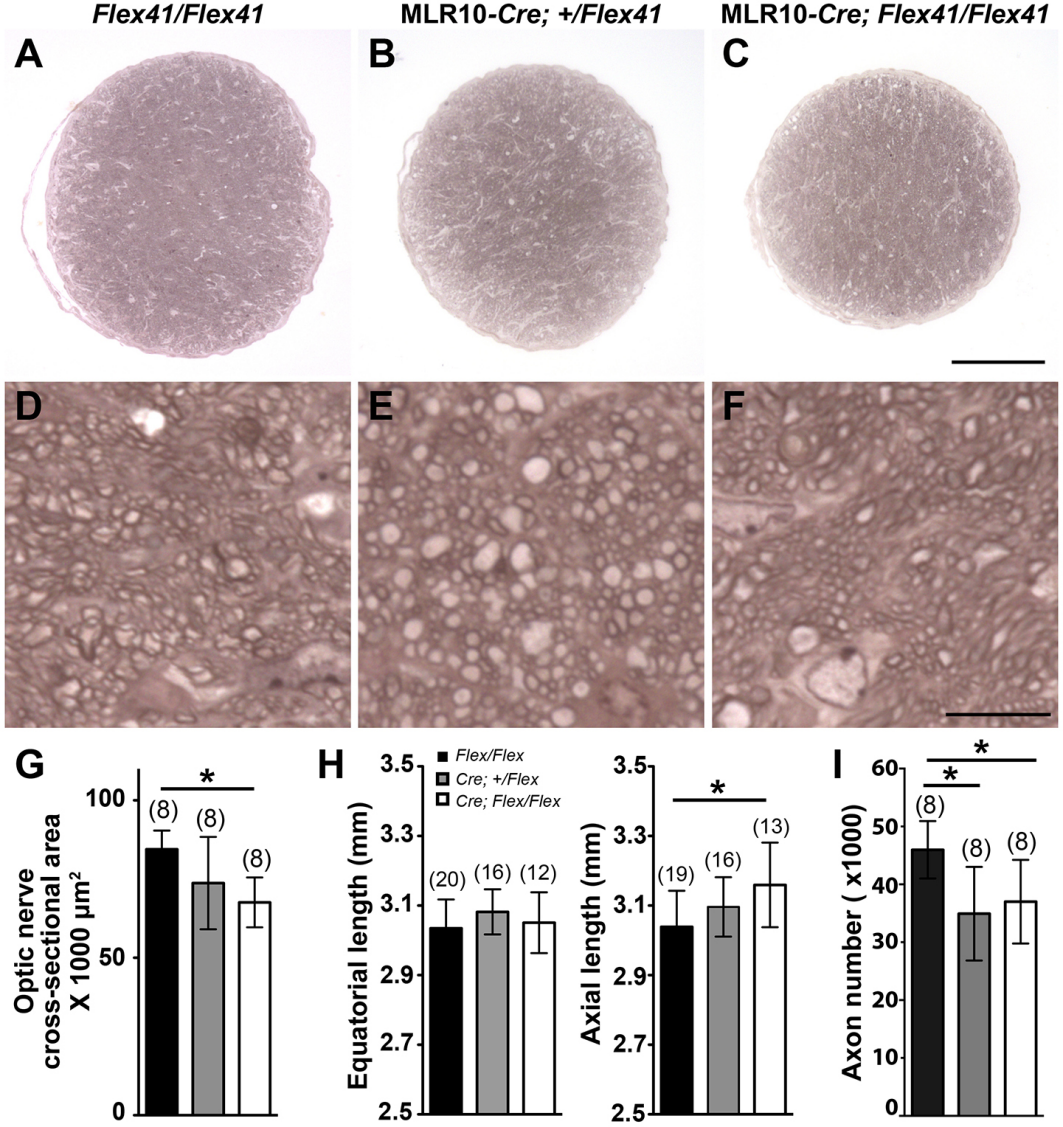
**Selective expression of mutant COL4A1 in lens leads to optic nerve hypoplasia.** (A-F) Optic nerves from lens-specific conditional mutants at 1.0-1.5 months of age sectioned and stained with PPD. Compared with controls (*Col4a1^Flex41/Flex41^*), the cross-sectional area of optic nerves from MLR10*^Cre^;Col4a1^Flex41/Flex41^* is smaller (A-C) whereas individual axons appear healthy (D-F) in all genotypes. Scale bar: 100 μm for A-C and 10 μm for D-F. (G) Quantification of optic nerve cross-sectional area. (H) Ocular sizes in 1.0- to 1.5-month-old mice assessed by measuring both the equatorial and axial lengths of control and conditional mutant eyes. While ocular equatorial lengths are comparable for all genotypes, axial lengths of MLR10*^Cre^;Col4a1^Flex41/Flex41^* eyes are slightly increased compared with controls, which is consistent with enlarged anterior chamber in these eyes. (I) Quantification of axon numbers in optic nerves from mice at 1.0-1.5 months of age. Both heterozygous and homozygous lens-specific conditional mutants show a significantly reduced number of myelinated, healthy axons compared with controls. Data in G-I are presented as mean±s.d. Numbers in parentheses indicate number of nerves or eyes analyzed. *P<0.05 by one-way ANOVA with Tukey's *post hoc* test.

We have shown previously that *Col4a1^+/Δex41^* mice maintained on a pure C57BL/6J genetic background have optic nerve hypoplasia (Gould et al., 2007). To test if the cell non-autonomous effects of lens-derived mutant COL4A1 extended to the optic nerve, we sectioned optic nerves from lens-expressing mutants at 1.0-1.5 months of age and stained them with PPD to distinguish between healthy and damaged axons (Fig. 5A-F). Optic nerves from MLR10*^Cre^*;*Col4a1^+/Flex41^* mice tended to have smaller cross-sectional areas compared with littermate controls (73,716.1±14,671.0 and 83,780.6±6069.4 μm^2^, respectively; mean±s.d., *P*=0.075) and nerves from MLR10*^Cre^;Col4a1^Flex41/Flex41^* mice were significantly smaller than controls (67,580.5±7916.9 μm^2^, *P*=0.00027) (Fig. 5G). To test whether reduced optic nerve cross-sectional area might be a consequence of smaller eyes, we measured the globe sizes using a caliper (Fig. 5H). We found no differences in equatorial lengths between genotypes; however, the axial lengths of MLR10*^Cre^;Col4a1^Flex41/Flex41^* eyes were slightly but significantly greater, consistent with increased depths of the anterior chambers that we detected by slit lamp biomicroscopy (Fig. 4H). Nevertheless, these results suggest that the reduced size of the optic nerve is not likely to be caused by overall growth retardation of mutant eyes. We previously showed that *Col4a1^+/Δex41^* mice have mislocalized retinal ganglion cells and increased apoptosis during retinogenesis (Labelle-Dumais et al., 2011). To test if optic nerve hypoplasia in lens-specific mutants might also be caused by a reduced number of retinal ganglion cells, we quantified the number of axons in the optic nerve. Although axons in both heterozygous and homozygous lens mutants were mostly healthy and myelinated, the total number was significantly reduced compared with controls (Fig. 5I). These findings suggest that, in addition to the effect on the development of the anterior segment, expression of mutant COL4A1 in the lens can influence optic nerve development.

### Lens conditional mutants have age-related IOP abnormalities and retinal damage

In some genetic contexts, *Col4a1^+/Δex41^* mice have chronically dysregulated IOPs and optic nerve damage that mimic glaucoma (Gould et al., 2007; Mao et al., 2015b) and *COL4A1* mutations can cause developmental glaucoma in patients (Sibon et al., 2007; Deml et al., 2014; Giorgio et al., 2015; Meuwissen et al., 2015; Plancher et al., 2015). To test if lens-specific expression of mutant COL4A1 caused age-related phenotypes, we aged a cohort of mice for 8-10 months. Slit lamp examination revealed that the anterior chamber became progressively larger in MLR10*^Cre^;Col4a1^Flex41/Flex41^* mice but not in mice of other genotypes (Fig. S4A-F). Moreover, the MLR10*^Cre^;Col4a1^Flex41/Flex41^* mice had abnormal IOP profiles (Fig. S4G) in which some eyes had high IOP (2 out of 12 had IOPs>21 mmHg) and others had very low IOP (3 out of 12 had IOPs<10 mmHg). Histological analysis revealed severe defects, including luxated lens, anterior synechia, ciliary body dysgenesis and pan-retinal thinning (data not shown) in three out of six eyes from MLR10*^Cre^;Col4a1^Flex41/Flex41^* mice. Examination of optic nerves and retinas from the other three eyes (Fig. S4H-P) showed mild, focal retinal disorganization (Fig. S4N-P) and thinning (data not shown) while the optic nerve heads were unremarkable (Fig. S4K-M). Histological analysis of optic nerves also showed that five out of eleven of the optic nerves were severely degenerated (Fig. S4J). Notably, abnormally low IOPs, severe retinal defects and severely damaged optic nerves were usually observed in the same eyes, suggesting that the optic nerve degeneration in these mice is not simply IOP-related retinal ganglion cell death but may be a consequence of other defects. It has been shown that primary lens defects and leaked lens material into the anterior chamber can cause inflammation and ocular pathology including uveitis and phacolytic glaucoma (Ellant and Obstbaum, 1992; Wilcock and Peiffer, 1987; Van Der Woerdt, 2000; Flocks et al., 1955; Yan et al., 2014). However, we did not observe macrophages or other inflammatory debris in the anterior chambers or the drainage structures of MLR10*^Cre^;Col4a1^Flex41/Flex41^* mice by histology (Fig. S4Q-S). We next labeled ocular sections from lens-expressing mutants at 1 month of age with antibodies against a major lens component, αB-crystallin (CRYAB), and a pan-macrophage marker, CD11b (Fig. S5), to evaluate the possibility of lens-induced ocular inflammation. While we did not detect differential CRYAB labeling between control and mutant eyes (Fig. S5A-D), the number of CD11b-positive cells increased in corneas from MLR10*^Cre^;Col4a1^Flex41/Flex41^* (Fig. S5E-H), supporting the possibility that inflammation might contribute to cell non-autonomous effects mediated by the lens. Notably, however, CD11b-positive cells were not detected in the ocular drainage structures or the anterior chamber.

We previously proposed that cell-autonomous lens defects might result from aberrant protein folding and intracellular accumulation of mutant COL4A1 (Gould et al., 2007). Protein misfolding can lead to ER stress that activates the unfolded protein response (UPR) pathway, and sustained ER stress and UPR activation can be detrimental to cells (Schröder and Kaufman, 2005). Although we detected abundant intracellular COL4A1 in lenses from *Col4a1^+/Δex41^* mice as early as E12.5 (Fig. S6A-B) we did not detect differential expression of UPR markers between *Col4a1^+/+^* and *Col4a1^+/Δex41^* eyes (Fig. S6C). We also used a genetic reporter line [ER stress activation indicator (*ERAI*)] (Iwawaki et al., 2004; Alavi et al., 2015) that produces a fluorescent protein (Venus) upon UPR activation to evaluate ER stress in eyes from postnatal *Col4a1^+/Δex41^* mice and did not detect evidence of UPR activation (Fig. S7). Therefore, while mutant COL4A1 accumulates at detectable amounts in lens epithelium (but not in corneal endothelium), the ability to detect subsequent ER stress is less robust.

### Key pathogenic events occur during early ocular organogenesis

**Fig. 6.**
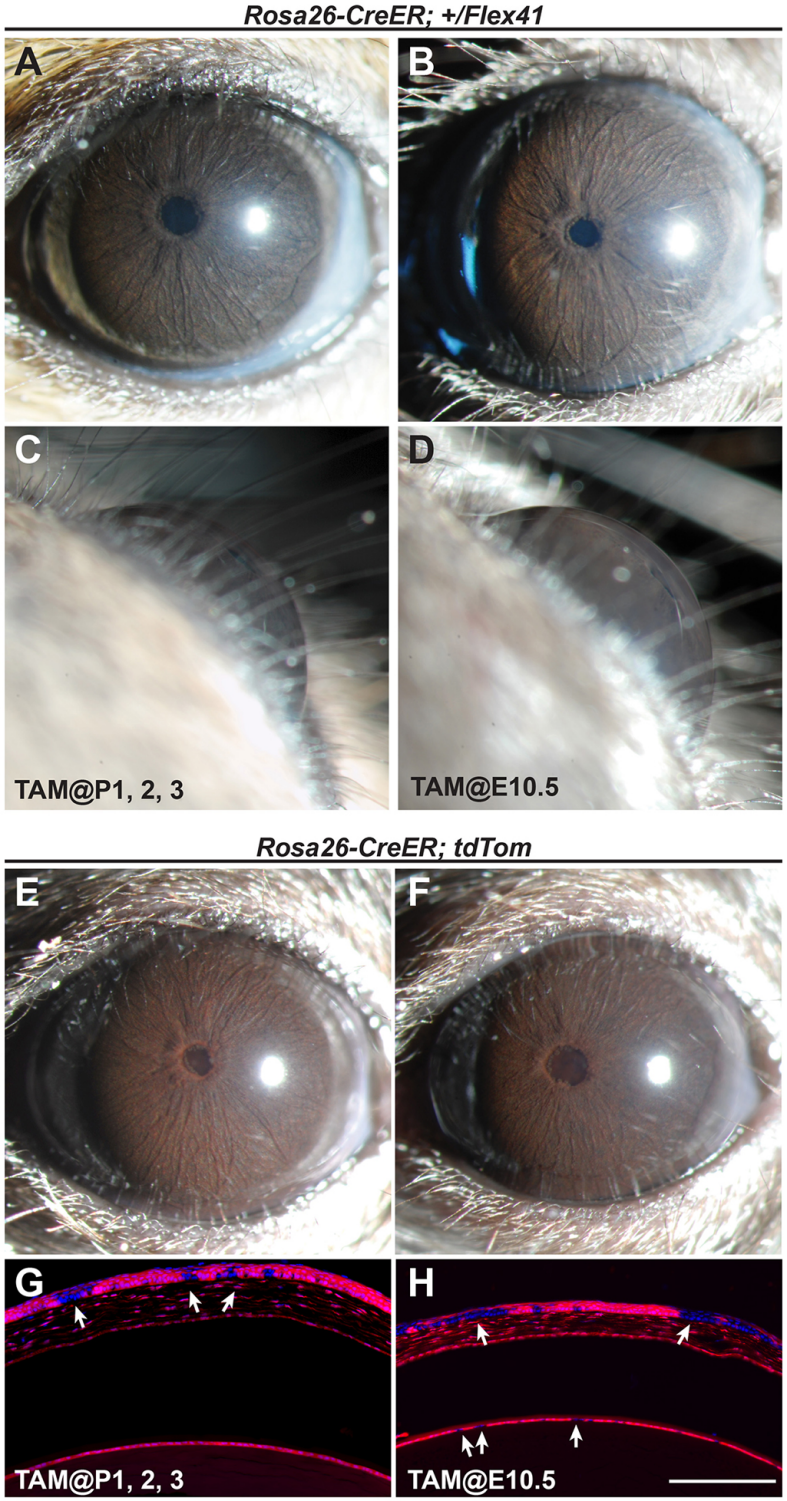
**Key pathological insults leading to COL4A1-related ASD occur during early embryonic development.** (A-D) *Col4a1^+/Flex41^* mice crossed to an inducible *Rosa26^Cre-ER^* strain for ubiquitous expression of mutant COL4A1 protein. Tamoxifen was injected at two different time points during development (P1-3 or E10.5) and recombination occurs hours later. Eyes were examined by slit lamp at 1.0-1.5 months of age. At both time points (*n*=24 and 10 eyes for P1-3 and E10.5), tamoxifen injection did not cause ASD, suggesting that key pathological events occurred prior to mid-gestation. (E-H) As a control for successful injection and CRE activation, the tdTomato CRE-reporter line was used. Expression of the red tdTomato protein causes a red/brown color of the iris and lens upon slit lamp examination (E,F). Cross-sections (G,H) of eyes from tamoxifen injected mice indicate patchy expression (white arrowheads) of tdTomato in the cornea and lens, indicating incomplete recombination.

To investigate the timing of pathogenesis, we crossed *Col4a1^+/Flex4^*^1^ mice with an inducible ubiquitous *Cre* line, *Rosa26^Cre-ER^* (Badea et al., 2003), in which CRE is activated after administration of tamoxifen. Since the majority of the anterior segment and drainage structures continue to develop for several weeks after birth (Smith et al., 2001), we initially injected tamoxifen at postnatal days (P) 1-3 (Fig. 6A,C); however, these eyes appeared to be normal. Next, we injected pregnant dams at E10.5 and, again, eyes from *Rosa26^Cre-ER^*;*Col4a1^+/Flex41^* mice appeared normal at 1.0-1.5 months of age (Fig. 6B,D). To determine whether insufficient CRE activation or recombination could explain the inability to detect a phenotype, we tested for recombination by RT-PCR and used the tdTomato reporter line to fluorescently label cells in which CRE was activated. We detected the presence of recombinant *Col4a1* mRNA in eyes from mice injected at E10.5 (Fig. S2B) and mice that were injected with tamoxifen postnatally (P1-3) or embryonically (E10.5) and aged to 1 month showed high levels of recombination with the reporter (Fig. 6E-H). Upon slit lamp examination, eyes were visibly red and histological sections of these eyes confirmed widespread recombination. However, unlabeled cells were also observed throughout ocular tissues and the recombination efficiency appeared to be lower in eyes from mice that were injected at E10.5. Taking into account the time required for CRE expression following Tamoxifen injection, these data suggest that the key pathogenic events likely occur at a time point earlier than E10.5-E12.5. However, COL4A1 secretion from cells that escape recombination could be compensatory and precludes a definitive conclusion.

## DISCUSSION

Our results indicate a critical role for the lens in ASD pathogenesis caused by a *Col4a1* mutation. Mice with lens-specific expression of mutant *Col4a1* had cataracts and exhibited a ‘dose-dependent’ pathology in non-lens tissues, including iridocorneal adhesions, enlarged anterior chambers, abnormal IOPs and small optic nerves, suggesting that important pathogenic mechanisms are cell non-autonomous. When we aged lens-specific mutants to determine if iridocorneal dysgenesis and adhesions might impact IOP we observed elevated IOP in a subset of mice and abnormally low IOP in others. Notably, the eyes with abnormally low IOP also had widespread retinal and optic nerve damage indicating that lens-specific expression of mutant *Col4a1* had broader consequences in ocular tissues that might be caused by secondary events including inflammation. Nonetheless, *COL4A1* mutations cause developmental glaucoma in patients (Sibon et al., 2007; Deml et al., 2014; Giorgio et al., 2015; Meuwissen et al., 2015; Plancher et al., 2015) and mice (Mao et al., 2015b) and the lens clearly contributes to *Col4a1*-related ASD in mice.

The importance of the lens in anterior segment development is well established (Beebe and Coats, 2000) and dysgenesis of ocular anterior segment structures are frequently observed in mouse models of lens-specific mutants (Choi et al., 2014; Flügel-Koch et al., 2002; Reneker et al., 2000) and in patients with mutations in genes with lens-specific expression (Semina et al., 2001, 1998; Jamieson et al., 2002). Here, we show the presence of CD11b-positive cells in eyes from lens-expressing mutants raising a possible contributory role of secondary inflammatory processes. Another possibility is that mutant COL4A1 impacts survival, migration or differentiation of periocular mesenchyme by perturbation of developmental pathways regulated by signaling molecules that are secreted by the lens (Ittner et al., 2005; Lwigale and Bronner-Fraser, 2009) or through direct contact as invading mesenchyme migrates over the surface of the lens (Cintron et al., 1983). Notably, lens removal from chick embryos caused dysgenesis of mesenchyme-derived tissues, which was preventable if the lens was replaced; however, correct lens orientation was required (Beebe and Coats, 2000). This observation suggests that diffusible signaling molecules might not be the only important way in which lens induces migration or differentiation of periocular mesenchyme. Our data do not distinguish between a secondary consequence of ocular inflammation or abnormal signaling and/or interaction between the lens and the periocular mesenchyme in the pathogenesis of *Col4a1*-related ASD; importantly these potential mechanisms are not mutually exclusive. Although ER stress has previously been associated with *Col4a1* mutations in certain tissues (Jones et al., 2016), when we tested markers of UPR using different techniques at different ages we did not detect evidence of UPR activation, despite of the presence of increased intracellular COL4A1 in the lens. Therefore, although ER stress may be present and the UPR activated in some circumstances, the direct significance of this pathway in pathogenesis is uncertain.

Heterozygosity for lens-specific expression of mutant *Col4a1* did not recreate the full *Col4a1*-related ASD phenotypic spectrum that has been described in *Col4a1^+/Δex41^* mice. Moreover, there was a dose-effect whereby homozygous mice exhibited more severe phenotypes. These observations may be explained by compensatory heterotrimer deposition by other cell types [for example, tunica vasculosa lentis or ciliary body (Sarthy, 1993; Dong et al., 2002)] and suggest that the full phenotypic manifestation results from the integration of independent pathogenic events in multiple tissues. Most of the tissues affected in ASD are derived from periocular mesenchyme, which is predominantly derived from neural crest cells with some contribution of cranial paraxial mesoderm. However, we did not detect a phenotype in mice that selectively expressed mutant *Col4a1* in neural crest-derived cells, suggesting that dysgenesis might occur from cell non-autonomous mechanisms. Unlike lens-specific conditional mutants, we did not test homozygous mutant animals and it is possible that a neural crest contribution would be unveiled in this setting. Although unlikely given their minimal contribution to the periocular mesenchyme, it is possible that COL4A1 deposition from mesodermal cells in the mosaic mesenchyme compensated and masked a contribution of neural crest cells. Notably, the endothelial lining of Schlemm's canal, a key component of the aqueous humor outflow pathway is primarily derived from mesoderm (Gage et al., 2005) and explicitly testing the contribution of these cells may warrant further consideration.

Selective expression of mutant *Col4a1* in vascular endothelial cells suggests a potential role for the vasculature in the etiology of ASD. Although our results using the *Col4a1^Flex4^*^1^ allele did not support a contribution of vascular endothelial cells in COL4A1-related ASD, selective expression of the *Col4a1^Flex41-neo^* allele in vascular endothelial cells leads to highly penetrant ASD as well as ONH. The *Col4a1^Flex41-neo^* allele had a mild baseline phenotype and could not be made homozygous, suggesting that this allele could behave as a null or hypomorphic mutation even in the absence of recombination (Holzenberger et al., 2000). One interpretation of these results is that there is a vascular contribution to ASD but that the pathogenic threshold was only achieved when vascular endothelial cell-specific expression of mutant COL4A1 was in a sensitized context generated by the *Col4a1^Flex41-neo^* allele. Moreover, vascular basement membranes are derived from multiple cell types, presenting an even greater potential for compensatory heterotrimer deposition from other sources, notably the pericytes and astrocytes. For instance, *Col4a1* mutant mice have severe cerebrovascular disease and intracerebral hemorrhages; however, selective expression of mutant *Col4a1* individually in vascular endothelial cells, pericytes or astrocytes each resulted in a very mild phenotype compared with that observed in *Col4a1^+/Δex41^* mutants (Jeanne et al., 2015). Therefore, it is likely that a full appreciation of the vascular contribution to ASD would require simultaneous combinatorial expression of mutant COL4A1 in other cell types that contribute to the vascular basement membrane in addition to vascular endothelial cells.

To define the timing of pathogenic events, we induced ubiquitous expression of mutant COL4A1 in early postnatal mice and during mid-gestation but, surprisingly, we did not observe pathology in either cohort. We confirmed tamoxifen delivery and CRE induction by using a reporter line and validated recombination of the conditional allele by RT-PCR. Considering the time required for *Cre* expression, recombination and expression of the conditional *Col4a1* mutant allele, this result may suggest that key pathogenic events occur before E10.5-12.5. However, there are technical limitations to these experiments that must be considered. For example, tamoxifen-induced CRE activation is not observed in all cells and the recombination efficiency was lower in embryos compared with postnatal mice. Moreover, recombination efficiencies are not equal at all loci and it is possible that recombination of the *Col4a1^Flex41^* locus is even less complete than is indicated by the reporter. While these data suggest that pathogenic events occur before mid-embryogenesis, we cannot exclude the possibility that the role for COL4A1 is later in development and that incomplete recombination results in enough extracellular COL4A1 to have compensatory effects.

Our study provides new insights into the location and timing of events important for *Col4a1* mutations to cause ASD. Collectively, our data indicate that the lens, acting both via cell autonomous and non-autonomous mechanisms, is a critical site for COL4A1-related ASD pathogenesis, identify a potential role for the vasculature in the etiology of ASD and suggest that the pathogenic events may take place prior to E12.5. Based on these parameters, future studies will seek to identify the developmental signaling pathways that are perturbed in periocular mesenchyme and the molecular mechanisms by which *Col4a1* mutations cause ASD and glaucoma.

## MATERIALS AND METHODS

### Animals

All experiments were compliant with the ARVO Statement for the Use of Animals in Ophthalmic and Vision Research and approved by the Institutional Animal Care and Use Committee at the University of California, San Francisco. *Col4a1^+/Δex41^* mice were originally identified in a mutagenesis screen conducted at The Jackson Laboratory and the ocular phenotypes have been previously described (Gould et al., 2005, 2007). The *Col4a1^Flex41^* conditional mutation was produced by InGenious Targeting Laboratory (Stony Brook, NY). The original conditional allele contains a *neo* selection cassette (Alavi et al., 2016; Jeanne et al., 2015) that caused a hypomorphic ocular phenotype (Fig. S1). The *neo* selection cassette was then deleted in embryonic stem cells. The conditional mutant allele was crossed to *Actb^Cre^* (Lewandoski et al., 1997) for ubiquitous CRE expression or to MLR10*^Cre^* (Zhao et al., 2004), *Wnt1^Cre^* (Danielian et al., 1998) and *Tie2^Cre^* (Kisanuki et al., 2001) mice for cell-type-specific CRE expression. We used *Rosa26^Cre-ER^* (Badea et al., 2003) for ubiquitous inducible CRE expression. In all cases, experimental mice were hemizygous for the respective *Cre* transgenes. We used *Rosa26^tdTomato^* reporter mice (Madisen et al., 2010) to validate CRE-mediated recombination. The *ERAI* reporter line for ER stress was previously described (Iwawaki et al., 2004; Alavi et al., 2015). All strains were iteratively crossed to C57BL/6J (B6) mice for at least five generations and both genders were used for each genotype.

### Tamoxifen administration

To induce CRE activation during embryonic or postnatal development, mice were injected with tamoxifen (Sigma-Aldrich T5648) solubilized in ethanol and diluted in corn oil (Sigma-Aldrich C8267). For embryonic activation, pregnant females received a single intraperitoneal injection of tamoxifen (0.1 mg/g) mixed with progesterone (0.05 mg/g, Sigma-Aldrich, P3972) to counteract the estrogen agonist effects of tamoxifen. Cesarean sections were performed on pregnant females at E19.5 and pups were reared with foster mothers. For postnatal activation, pups received one intragastric injection of tamoxifen (50 μg) for three consecutive days starting from P1.

### Slit lamp biomicroscopy

Ocular anterior segment examinations were performed using a slit lamp biomicroscope (Topcon SL-D7; Topcon Medical Systems, Oakland, NJ) attached to a digital SLR camera (Nikon D200; Nikon, Melville, NY).

### Histological analyses

#### Eyes

Eyes were harvested at appropriate ages and fixed with 2% PFA and 2.5% glutaraldehyde in 0.1 M phosphate buffer, pH 7.4 overnight, dehydrated in graded ethanol and embedded in Technovit 7100 Glycol Methacrylate (Electron Microscopy Sciences, Hatfield, PA). Embedded tissues were sectioned and stained with Hematoxylin and Eosin (H&E).

#### Optic nerves

Post-orbital portions of optic nerves were processed and analyzed as previously described (Anderson et al., 2006). Briefly, the top of the skull and most of the brain overlying the optic nerves were removed and the remaining tissue was fixed overnight with 2% PFA and 2.5% glutaraldehyde in 0.1 M phosphate buffer, pH=7.4. Optic nerves were dissected, plastic sections were prepared by the UCSF VAMC Pathology Core and 1 μm cross-sections were stained with paraphenylenediamine (PPD), which differentially stains the axoplasm of sick or dying axons darkly, thus permitting detection of axonal injury. Cross-sectional areas of optic nerves were measured using Fiji software (Schindelin et al., 2012). The number of axons in optic nerves was quantified as previously described (Mao et al., 2011). Briefly, images from 18 non-overlapping fields that were evenly distributed throughout the optic nerve were taken at 1000× magnification. The axon number in each image was counted from a central area with a size that equals 1/180 of the total cross-sectional area of the optic nerve. The total axon number equals to the sum of axon counts from all 18 images multiplied by 10.

### Eye size measurement

Ocular axial (anterior to posterior) and equatorial (nasal to temporal) lengths were measured as previously described (Nair et al., 2011). Briefly, eyes were enucleated from mice after euthanasia. After removing fat and connective tissues surrounding the eye in PBS, eyes were placed and oriented using a dissecting microscope. A Vernier caliper (Fowler Ultra-Cal Mark III, Newton, MA) was used to measure the eye sizes under the microscope. Each data point was averaged from two consecutive measurements, and those with a standard deviation between two measurements larger than 0.1 mm were excluded. Two out of 98 data points were discarded in total.

### IOP measurement

Mice were anaesthetized with a steady flow of 2% isoflurane in oxygen. As soon as the respiratory rate fell between 1.0 and 1.4 times/s, IOP was measured using a rebound tonometer (TonoLab; Colonial Medical Supply, Franconia, NH). All measurements were taken between 12:00 h and 16:00 h. Each eye had at least three consecutive measurements a day (each recorded measurement was averaged from 6 readings), and a total of at least 9 measurements for each eye were taken on 3 separate days.

### Immunofluorescence labeling

Heads of E12.5 embryos or P1 pups were fixed in 4% paraformaldehyde overnight, cryoprotected in 30% sucrose/PBS, and embedded in optimal cutting temperature (Sakura Finetek, Torrance, CA). Then, 12 μm sections were prepared using a Leica CM1900 cryostat (Rankin Biomedical Corp. Holly, MI). To visualize tdTomato fluorescent signal, slides were counterstained with DAPI and images were acquired using an AxioImager M1 microscope equipped with an AxioCam MRm digital camera and AxioVision software (Carl Zeiss Microscopy). Alternatively, slides were incubated with 0.1 M KCl/HCl, pH 1.5 for 10 min at room temperature for antigen retrieval, blocked in PBS with 0.2% Triton X-100, 10% normal donkey serum and 1% BSA, and incubated in a rat COL4A1 antibody [1:100, H11 clone; Shigei Medical Research Institute, Okayama, Japan (Sado et al., 1995)] and rabbit Laminin 1+2 (1:500, ab7463, Abcam, Burlingame, CA, USA) at 4°C overnight. Other antibodies used include a goat anti-COLIV antibody (1:200, 1340-01, Southern Biotech, Birmingham, AL, USA) and a goat anti-GFP antibody (1:250, ab6673, Abcam). Following washes, slides were incubated in species-specific Alexa Fluor 488- or 594-conjugated secondary antibodies (Invitrogen) and nuclear DAPI stain, and images were acquired with a Zeiss LSM700 with Plan-Apochromat 63×/1.40 objective and ZEN software (Carl Zeiss Microscopy). At 1 month, eyes were enucleated, processed and sectioned as described above. After blocking, sections were incubated with a mouse monoclonal CPTC-CRYAB-3 antibody [1:150, Developmental Studies Hybridoma Bank (DSHB)] against αB-crystallin and a rat CD11b antibody (1:100, 550282, BD Pharmingen, CA, USA) followed by species-specific antibodies and nuclear DAPI stain and imaged with the Zeiss LSM700 microscope.

### RT-PCR

Total RNA was isolated from whole eyes using the RNAeasy Mini Kit (Qiagen, Valencia, CA) and reverse transcribed into cDNA using iScript Reverse Transcriptase Supermix (Bio-Rad, Hercules, CA). We performed PCR using primers in exon 40 and 42 of *Col4a1* (Forward, 5′-TCGGCAGGAGAGAAGGGT-3′; Reverse, 5′-GCCAGGTAAGCCAGGTTG-3′) to amplify 195 bp or 144 bp fragments from wild-type and recombinant forms of *Col4a1*, respectively.

### Quantitative PCR (qPCR)

Total RNA from E14.5 lenses was collected with an RNA micro kit (Qiagen) and reverse transcribed into cDNA. Quantitative PCR was performed using the SsoFast Evagreen mix (Bio-Rad) on a Bio-Rad CFX96. Primer sequences include: *Hspa5*, 5′-ACCCCGAGAACACGGTCTT-3′ and 5′-GCTGCACCGAAGGGTCATT-3′; *Atf6*, 5′-TTTGATGCCTTGGGAGTCAG-3′ and 5′-GATGGAGCAACTGGAGGAAG-3′; *Atf4*, 5′-GCAAGGAGGATGCCTTTTC-3′ and 5′-GTTTCCAGGTCATCCATTCG-3′; *Ddit3*, 5′-GTCCCTAGCTTGGCTGACAGA-3′ and 5′-TGGAGAGCGAGGGCTTTG-3′; *Xbp1s*, 5′-GAGTCCGCAGCAGGTG-3′ and 5′-GTGTCAGAGTCCATGGGA-3′; *Herpud1*, 5′-ACAAAGGGTGCTGAATCCAC-3′ and 5′-CCTTGGAAAGTCTGCTGGAC-3′; *Dnajb9* 5′-TAAAAGCCCTGATGCTGAAGC-3′ and 5′-TCCGACTATTGGCATCCGA-3′; and *Hprt1* 5′-TGACACTGGCAAAACAATGCA-3′ and 5′-GGTCCTTTTCACCAGCAAGCT-3′*. Hprt1* was used as an internal normalization control. Relative expression was calculated based on the 2^−ΔΔCT^ method (Livak and Schmittgen, 2001).
